# Alterations of gut virome with close interaction in the progression of estrogen deficiency-induced osteoporosis

**DOI:** 10.1080/19490976.2024.2437250

**Published:** 2024-12-09

**Authors:** Yueqi Chen, Chuan Yang, Zihan Deng, Tingwen Xiang, Jiulin Tan, Jianzhong Xu, Dong Sun, Fei Luo

**Affiliations:** a Department of Orthopedics, Southwest Hospital, Third Military Medical University (Army Medical University), Chongqing, People’s Republic of China; b Department of Orthopedics, Chinese PLA 76th Army Corps Hospital, Xining, People’s Republic of China; c Department of Biomedical Materials Science, Third Military Medical University (Army Medical University), Chongqing, People’s Republic of China

**Keywords:** Osteoporosis (OP), gut virome, metagenomics, gut microbiota homeostasis, bacteriome–virome interaction

## Abstract

Previous research has established a link between gut microbiota and osteoporosis (OP) advancement. However, there remains a limited understanding of the crucial contribution of the gut virome in the onset and progression of OP. We employed metagenomic shotgun sequencing and gut virome sequencing to process the ovariectomy (OVX)-induced OP murine model, which revealed significant disparities in bacteriome and virome compositions between subjects with OP and healthy controls. One hundred and seventy-four altered viral strains were identified to participate in the multifaceted regulation of bone loss, involving immune modulation, microbial metabolic activity, and intricate host-virus dynamics. Our findings suggested that the gut virome may influence bone metabolism, potentially altering the balance of bone-modulating compounds like short-chain fatty acids. This comprehensive analysis of the gut virome in OP highlighted the diagnostic potential of combined gut viral and bacterial biomarkers for OP.

## Introduction

Various microorganisms colonize the human digestive tract from birth, and its diversity and composition shift over time in correlation with the host’s anatomy, diet, and specific nutritional requirements.^1^ The human gut microbiome extensively affects multiple metabolic processes, including the digestive, endocrine, immune, and skeletal systems, and its disruption contributes to diseases ranging from digestive, cardiovascular, respiratory, and metabolic disorders to cancers.^2,3^ Indeed, gut microorganisms comprise bacteria, fungi, archaea, and viruses, which reside within the human digestive tract and have been identified as regulators of intestinal equilibrium.^4^ Previous studies involved in gut microorganisms focused on gut bacteria; increasing attention is devoted to the viral components of the gut microbiome, referred to as the “gut virome.”

The prokaryotic-infecting viruses (bacteriophages) are viruses that infect bacteria and dominate in the composition of gut virome.^5,6^ These bacteriophages are enormously diverse, harboring a myriad of genetic material and sharing both lytic and lysogenic life cycles.^7^ In the lytic cycle, bacteriophages infect host bacteria, replicate within them, and ultimately cause the bacterial cell to lyse, releasing new viral particles. Conversely, during the lysogenic cycle, the bacteriophage genome, which is referred as a prophage, is incorporated into the host genome, where it may remain dormant for an extended period or provide specific advantages to the host, such as conferring resistance to other phages or toxins.^8,9^ Bacteriophages play a role in molding microbial populations through ongoing “predator-prey interactions” and horizontal gene transfer (from viruses to bacteria), consequently impacting the health and disease of the host.

Specifically, this bacteriophages–bacteria interaction shapes the diversity and functionality of host gut microbiota, integrating bacteriophages into a complex symbiotic nexus encompassing both microflora and host entities. For instance, *Danielle E Campbell et al*. reported that the temperate phage, *Bacteroides phage BV01*, represses the tryptophan-rich sensory protein (TspO) in *Bacteroides vulgatus* and thereby potentially impacts host bile acid signaling.^10^ *Adam G. Clooney et al*. demonstrated that in the gut virome of healthy individuals, there exists a consistent set of virulent bacteriophages, which transition to temperate bacteriophages in cases of inflammatory bowel disease (IBD). This alteration is associated with a decline in bacterial diversity.^11^ Additionally, fructose and short-chain fatty acids (SCFAs) could activate the Ack pathway in gut symbiont *Lactobacillus reuteri*, which is related to acetic acid production, and in turn initiates the bacterial stress response that enhances bacteriophage replication and leads to lysis of their bacterial hosts.^12^ Furthermore, the gut virome communicates closely with the host’s immune system, playing a vital role, particularly in intestinal immunity. For instance, bacteriophages could be enriched in mucosal surfaces by binding interactions between mucin glycoproteins and Ig-like protein domains exposed on phage capsids, thereby limiting mucosal bacteria and protecting these epithelial barriers from bacterial infection and translocation.^13^ In this context, diverse phages may stimulate immune cells, leading to subsequent immune responses. As previously reported, the disruption of commensal bacteriophage populations could result in immune response dysregulation, which promotes the progression of multiple chronic immunological disorders, including IBD, obesity, and rheumatoid arthritis (RA).^14–16^ Alterations in the gut virome have not only been associated with diseases in the gut, such as IBD and colorectal cancer, but also with the development of diseases in distant organs, including pulmonary arterial hypertension, Parkinson’s disease, nonalcoholic fatty liver disease, and type 2 diabetes (T2D).^17^ Generally, gut virome profoundly affects the host’s immune response, metabolism, and overall health. However, the specific role of the gut virome in the pathogenesis of certain diseases remains poorly understood.

Here, we reported gut virome alterations in estrogen deficiency-induced OP. OP is one of the most widespread bone diseases in women post-55 and men post-65, resulting in bone fragility and susceptibility to fracture.^18^ Research in epidemiology has shown that OP affects 10.2% of people over 50, and projections suggest this figure will increase to 13.6% by 2030, leading to a significant strain on global healthcare systems.^19^ Multiple factors, including aging, genetic variations, medication intervention as well as estrogen deficiency, have been proven to play a vital role in OP progression. While extensive research has been conducted on the role of gut microbiota in various diseases, such as OP, the role of gut virome in this area has not been thoroughly examined.^20^

In our study, we aimed to investigate the alterations in the gut virome and map the intimate association between gut virome and the progression of estrogen deficiency-induced OP. Utilizing high-throughput metagenomic shotgun sequencing and virome sequencing techniques, we assessed the viral diversity and identified key viral components within the gastrointestinal tract, which may provide novel insight into the diagnosis, treatment, and preventive intervention of OP.

## Results

### Alterations of gut bacteriome in OVX-induced OP murine model

**Figure 1.**
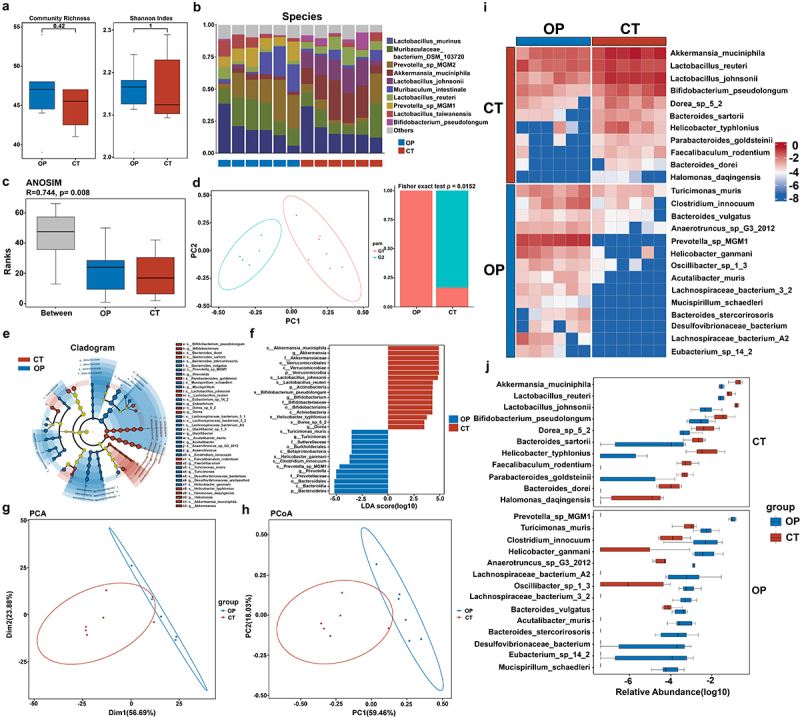
The profiles of gut bacteriome between OP and CT groups.

It is known that bacteria are one of the most abundant and diverse microbial communities in the human body, which widely participates in the modulation of overall gut homeostasis.^21^ These bacterial populations perform as hosts for a diverse array of bacteriophages, the viruses that specifically infect bacteria. Concretely speaking, the bacterial component critically shapes the virome by providing the ecological niches and the physical substrates required for phage replication. Therefore, we established a murine model of OP induced by OVX to systematically evaluate the alterations in gut bacteriome via metagenomic shotgun sequencing. Initially, we assessed gut microbial diversity using alpha diversity indices, including Community Richness and the Shannon Index, which demonstrated no significant differences between the OP and CT groups (Figure 1a). This indicated that estrogen deficiency did not markedly alter the overall diversity of the gut microbiota in terms of richness and evenness. To provide an intuitive depiction of the species composition within each group, we presented a taxonomic bar chart at the species level that showcased the top 10 most abundant species and arranged the samples in descending order of the most abundant species within the group (Figure 1b). Subsequently, ANOSIM (Analysis of Similarities) of species abundance revealed a significant distinction in microbial profiles between the OP and CT groups (*R* = 0.744, *p* = 0.008) (Figure 1c). Moreover, Partitioning Around Medoids (PAM) clustering analysis of the gut microbiota communities illustrated that the distribution of gut microbial species between the OP mice and the healthy controls is markedly different. The Fisher exact test demonstrated that the proportions of taxa within each group displayed significant differences (*p* = 0.0152) (Figure 1d).

(a) Boxplots of related alpha diversity consisting of Community Richness and Shannon Index.

(b) Bar graphs of the bacteria at species taxonomic level between OP and CT groups.

(c) Analysis of similarities at the species level.

(d) PAM clustering PCoA analysis at the species level. Fisher exact test was used to check whether there was any difference in the proportion of classification in each group.

(e) A phylogenetic tree with cladogram computed by linear discriminant analysis effect size (LEfSe) algorithm depicted taxonomic association between microbiome communities from OP and CT groups. The circles radiating from inside to outside represented the taxonomic level from Phylum to Species. Each node on different levels represented the specific taxon, and the node diameter was proportional to the relative abundance. Yellow nodes represented no significant difference in species between the two groups. Red nodes indicated taxa predominant in CT mice. Blue nodes indicated taxa predominant in OP mice.

(f) LDA score indicated statistical differences in species between OP and CT groups.

(g) Principal Component Analysis between OP and CT groups.

(h) Principal Co-ordinates Analysis based on Bray-Curtis distances at the species level.

(i) Heatmap of different gut microbiota at species taxonomic level between OP and CT groups. Color in the heatmap was utilized to describe specific species’ abundance, with blue indicating lower abundance and red indicating higher abundance.

(j) Statistical differences in gut microbiota at the species taxonomic level between OP and CT groups were evaluated with box plots.

To further identify the specific microbial taxa that exhibited the most significant differences between OP and CT groups and were associated with OP progression, we performed LEfSe (linear discriminant analysis Effect Size) and Cladogram (based on maximum relative abundance difference in each level) analyses. As shown in Figure 1e,f, we determined many differentially abundant taxa at the phylum level: *Bacteroidetes* was enriched in the OP group, whereas *Actinobacteria* and *Verrucomicrobia* were predominant in the CT group (The criteria for selection included an LDA score greater than 2 and a P-value less than 0.05). Specifically, 13 differential taxa were detected at the genus level, with 5 enriched in the CT group and eight in the OP group. At the species level, we found 26 differential taxa – 11 enriched in the CT group and 15 in the OP group. Consistently, the principal component analysis (PCA) and principal coordinate analysis (PCoA; R^2^ = 0.46, *p* = 0.007) also showed a significant separation of the OP group and healthy controls (Figure 1g,h). Ulteriorly, we compared the differences in gut microbiota between the OP group and the control group at the species level, retaining species with a prevalence of no less than 10% in the population (Figure 1l,j). Notably, several pathogens (e.g., *Clostridium_innocuum* and *Helicobacter_ganmani*) were substantially enriched, and multiple probiotics (e.g., *Akkermansia_muciniphila*, *Lactobacillus johnsonii*, *Parabacteroides_goldsteinii*, and *Bifidobacterium_pseudolongum*) were notably reduced in OP subjects when contrasted with healthy controls, aligning with findings from other studies.^22–27^ Collectively, these results demonstrated a concurrent dysregulation in the composition of gut bacteria with the progression of OP.

### Diversity and composition alterations of the gut virome in OVX-induced OP murine model

**Figure 2.**
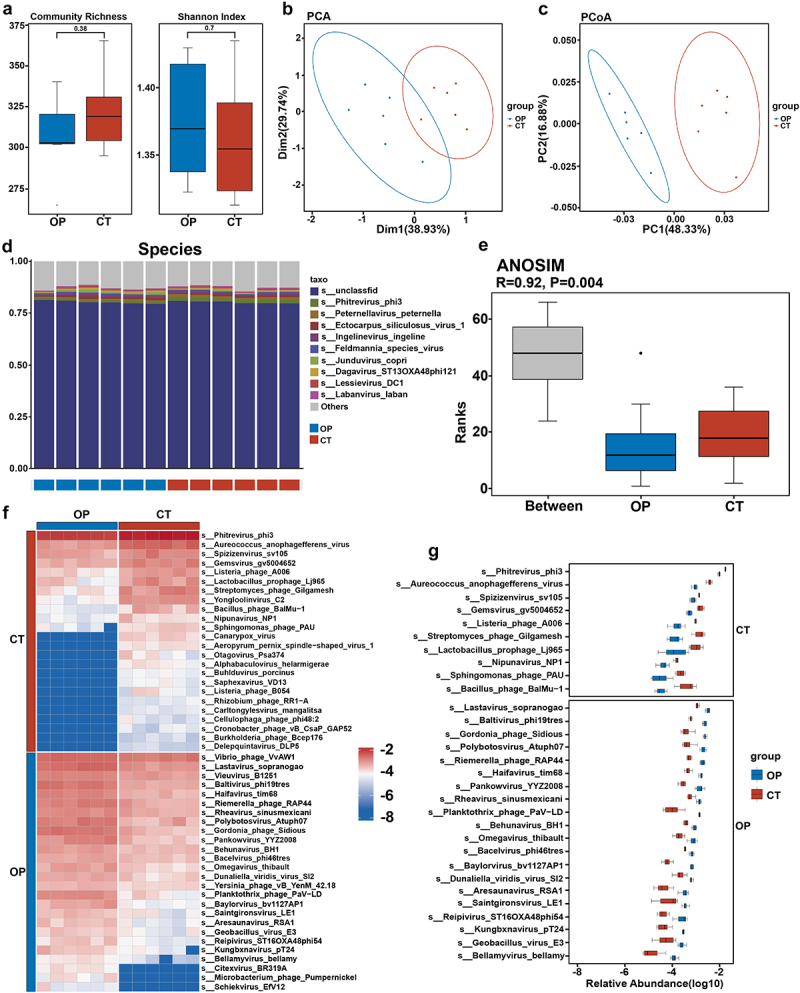
The profiles of gut virome between OP and CT groups.

Subsequently, we continued to investigate the complex microbial alterations in gut virome associated with estrogen deficiency-associated bone loss. We aimed to illuminate the multifaceted network of interactions within the microbiome that could be pivotal in estrogen deficiency-induced OP. Alpha diversity refers to the quantification of the variety and abundance of viral species, which can reflect the richness and diversity of the viral community. We assessed the alpha diversity through Community Richness and the Shannon Index and revealed no apparent difference between the OP and CT groups, suggesting a consistent level of species richness and evenness within the viral communities of both sets of subjects (Figure 2a). Beta diversity focuses on the comparison of viral communities between different groups, measuring the differences in composition and abundance of species. As depicted in Figure 2b, PCA analysis elucidated clear segregation between the OP and CT groups. Consistently, PCoA based on the Bray-Curtis distance showed that OP and CT groups were significantly divided into two distinct clusters (R^2^ = 0.453, *p* = 0.004, Figure 2c). These results indicated distinctive gut virome profiles despite the similar alpha diversity levels.

(a) Boxplots of related alpha diversity consisting of Community Richness and Shannon Index.

(b) Principal Component Analysis of gut virome between OP and CT groups.

(c) Principal Co-ordinates Analysis of gut virome based on Bray-Curtis distances at the species level.

(d) Bar graphs of the viruses at species taxonomic level between OP and CT groups.

(e) Analysis of similarities at the species level.

(f) Heatmap of different gut virome at species taxonomic level between OP and CT groups. Color in the heatmap was utilized to describe specific species’ abundance, with blue indicating lower abundance and red indicating higher abundance.

(g) Statistical differences of gut viruses at the species taxonomic level between OP and CT groups were evaluated with box plots.

In addition, we observed significant differences in gut virome between OP and CT subjects at genus and phylum levels via ANOSIM analyses (Fig. S1A and S1C). Specifically, at the genus level, *Phaeovirus*, *Fernvirus*, and *Freyavirus* dominated the two groups (Fig. S1B). At the phylum level, *Uroviricota*, *Nucleocytoviricota*, and *Peploviricota* accounted for the majority of identified virus sequences, indicating their dominant presence in the ecosystem (Fig. S1D). As shown in Fig. S2A and S2B, we noticed 180 markedly altered virus genera, including 108 genera enriched in OP mice and 72 genera enriched in the CT group. Furthermore, at the phylum level, *Nucleocytoviricota*, *Peploviricota*, *Pisuviricota*, and *Preplasmiviricota* were significantly upregulated, and *Lenarviricota* as well as *Uroviricota* were downregulated in OP mice (Figure 2c,d). The analyses of NMDS (Non-metric Multidimensional Scaling), PCA, and PCoA further displayed significantly different clusters, demonstrating dramatic differences in virus composition between the two groups at genus and phylum levels (Fig. S3A-S3F).

Species-level abundances were visually represented in Figure 2d, focusing on the top 10 species regarding average abundance, primarily *Phitrevirus_phi3* and *Peternellavirus_peternella*. Furthermore, a highly significant ANOSIM analysis result, with *R* = 0.92 and *p* = 0.004 shown in Figure 2e, confirmed the potential biological relevance of virome changes in OP. We dived into the specifics of differential species abundances by submitting a subset of species with at least 10% prevalence in the cohort to the Wilcox test to identify statistically meaningful differences. One hundred and seventy-four viral species were identified to be significantly altered in OP mice, of which 85 were predominant in the CT group and 89 enriched in the OP group. Notably, 30% of them are phages. Based on the abundance analysis of the altered viral strains, we found that *Phitrevirus_phi3* was most enriched in the CT group, while *Lastavirus_sopranogao* was most enriched in the OP group. These findings highlighted the significant differences in the viral compositions between healthy and osteoporotic conditions. The differential enrichment of these viral strains suggested their potential roles in the multifaceted regulation of bone metabolism. *Phitrevirus_phi3*, predominant in the CT group, may maintain a healthy bone state by possibly supporting beneficial microbial interactions and metabolic activities that promote bone health. On the other hand, *Lastavirus_sopranogao*, predominant in the OP group, might contribute to bone loss through mechanisms such as immune modulation or altering the microbial metabolic activities detrimental to bone density (Figure 2f,g). These findings uncovered substantial and intricate changes in the gut virome of subjects with OP, indicating that mice with OP exhibited pronounced gut viral dysbiosis, characterized by changes in both the diversity and taxonomy of viruses present.

### Functional alterations of gut bacteriome in OVX-induced OP murine model

**Figure 3.**
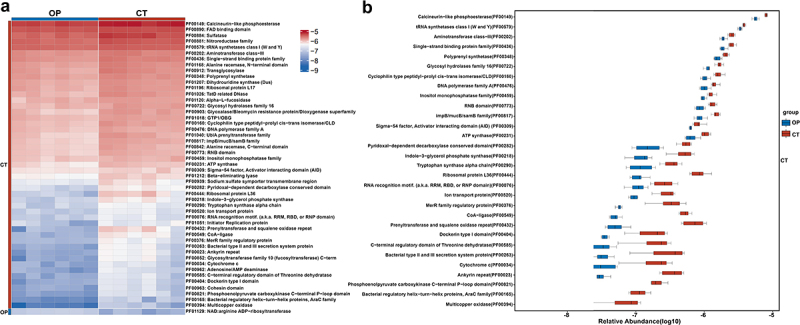
Functional alterations of gut bacteriome between OP and CT groups.

In exploring the functional dynamics of the gut microbiome in OVX-mediated OP mice, we assessed the anticipated bacterial activity of OP mice with that of healthy subjects. As shown in the heatmap, the OP group demonstrated loss of multiple bacterial functions in terms of Pfam protein functions (Figure 3a). Specifically, compared with the CT group, 1615 bacterial functions were identified to be downregulated in the OP group for the Pfam database. These down-regulated functions included Calcineurin-like phosphoesterase, tRNA synthetases class I (W and Y), Aminotransferase class−III, Single-strand-binding protein family, Polyprenyl synthetase, Glycosyl hydrolases family 16, Cyclophilin type peptidyl−prolyl cis−trans isomerase/CLD, DNA polymerase family A, Inositol monophosphatase family, and RNB domain (Figure 3b).

(a) Comparative heatmap of Pfam protein function distribution in the gut bacteriome of OP and CT groups.

(b) Boxplot analysis of Pfam protein function variability in the gut bacteriome of OP and CT groups.

The downregulation of these bacterial functions in the OP group demonstrated several critical aspects of gut microbiome alterations associated with estrogen deficiency-induced OP. For instance, calcineurin-like phosphoesterase plays a pivotal role in cellular signal transduction and its reduced activity could imply compromised regulatory pathways within the gut microbiome. The tRNA synthetases class I (W and Y) are essential for protein synthesis, and their downregulation suggested a potential decline in overall protein biosynthesis capacity, affecting microbial growth and function. Aminotransferase class-III is involved in amino acid metabolism and is crucial for the synthesis and degradation of amino acids. Reduced levels of this enzyme can lead to imbalances in nitrogen metabolism, potentially impacting the nutritional status of the host. The Single-strand binding protein family is vital for DNA replication and repair. Lower expression levels of these proteins may increase genomic instability within the gut microbiota. Polyprenyl synthetase is necessary for the synthesis of polyprenyl lipids, which are critical components of cell membranes and signaling molecules. A reduction in this enzyme may disrupt membrane integrity and cellular communication. Glycosyl hydrolases family 16 plays a significant role in carbohydrate metabolism, and its downregulation could lead to impaired digestion and utilization of complex carbohydrates, affecting the host’s energy balance. Inositol monophosphatase family is involved in phosphoinositide metabolism, which is crucial for cell signaling and membrane dynamics. Lower enzyme levels may impair intracellular signaling pathways within the gut microbiome.

Overall, the downregulation of these functions displayed the significant impact of estrogen deficiency on the gut microbiome’s ability to maintain essential biochemical processes, potentially contributing to the progression of OP through altered microbial-host interactions.

### Altered gut virome functions in a murine model of OP induced by OVX

**Figure 4.**
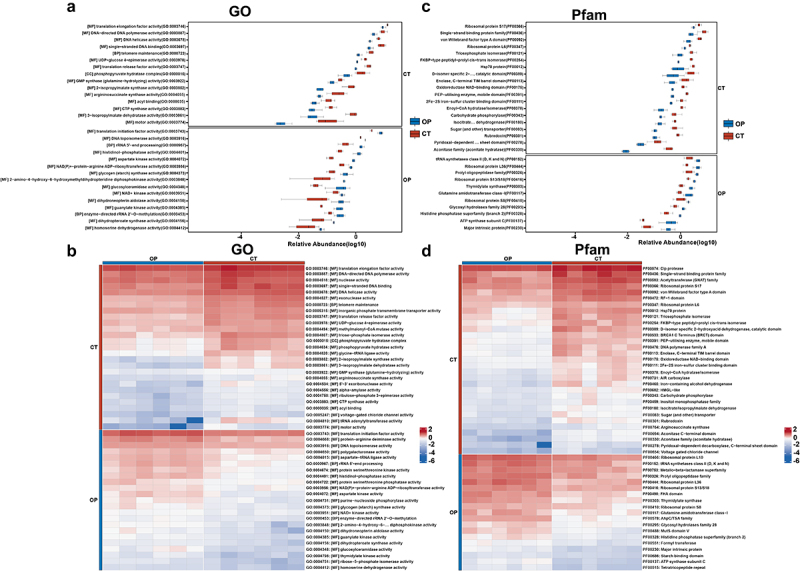
Functional alterations of gut virome between OP and CT groups.

To elucidate the potential functional implications of the virome alterations observed in the OP and CT groups, we performed comprehensive in silico analyses using the Pfam and the Gene Ontology (GO) resource. We initially compared the predictive gut virome function of OP mice with that of healthy controls via the GO database and identified 789 markedly altered viral functions (Figure 4a,b), among which 398 were lost, and 391 were enriched in OP mice. Notably, these downregulated viral functions were involved in translation elongation factor activity, DNA-directed DNA polymerase activity, DNA helicase activity, single-stranded DNA binding, UDP-glucose 4-epimerase activity, translation release factor activity, phosphopyruvate hydratase complex, GMP synthase (glutamine-hydrolyzing) activity, argininosuccinate synthase activity, CTP synthase activity, which was associated with the synthesis and processing of viral proteins and nucleic acids. These results suggested that gut viruses may lose practical replication ability, leading to less production of necessary proteins for assembling new virions. Interestingly, we noticed that translation initiation factor activity, DNA topoisomerase activity, rRNA 5’-end processing, histidinol-phosphatase activity, aspartate kinase activity, NAD(P)+-protein-arginine ADP-ribosyltransferase activity, glycogen (starch) synthase activity, 2-amino-4-hydroxy-6-hydroxymethyldihydropteridine diphosphokinase activity, and glucosylceramidase activity were upregulated in OVX mice, which were related to the modulation of DNA topology, maturation of ribosomal RNA, amino acid biosynthesis, energy metabolism, post-translational modification, polysaccharide biosynthesis, tetrahydrobiopterin metabolism, and glycosphingolipid degradation, respectively. Moreover, enhanced glycogen synthase activity might change the energy reserves of the host cell, affecting the energy-dependent steps of viral infection.

(a) Gene Ontology (GO) boxplot distribution of gut virome in OP and CT groups.

(b) GO heatmap for gut virome comparison between OP and CT groups.

(c) Boxplot analysis of Pfam protein function variability in the gut virome of OP and CT groups.

(d) Comparative heatmap of Pfam protein function distribution in the gut virome of OP and CT groups.

Consistently, we also identified multiple altered virome functions in the Pfam database, including 645 upregulated and 742 downregulated functions in OVX mice. As shown in Figure 4c,d the OP group demonstrated loss of multiple virome functions such as ribosomal protein S17, single-strand binding protein family, von Willebrand factor type A domain, ribosomal protein L6, triosephosphate isomerase, FKBP-type peptidyl-prolyl cis-trans isomerase and Hsp70 protein, which participate in essential biochemical pathways and cellular maintenance mechanisms. The observed functional diminishment within the OP group demonstrated significant alterations to the overall intestinal homeostasis. Given these proteins’ important role in maintaining cellular and viral life cycles, their reduced expression could impede viral particles’ proper assembly and function. Furthermore, it could hamper the defensive response of the host cells against viral infections by disrupting the normal protein synthesis pathways, cellular stress mechanisms, and potential immunomodulatory responses. This imbalance may contribute to dysbiosis and a concomitant shift in the intricacies of the gut microbiota–virome interactions, potentially augmenting susceptibility to enteric pathologies.

### Symbiotic and pathogenic associations in the gut microbiome during OP progression

**Figure 5.**
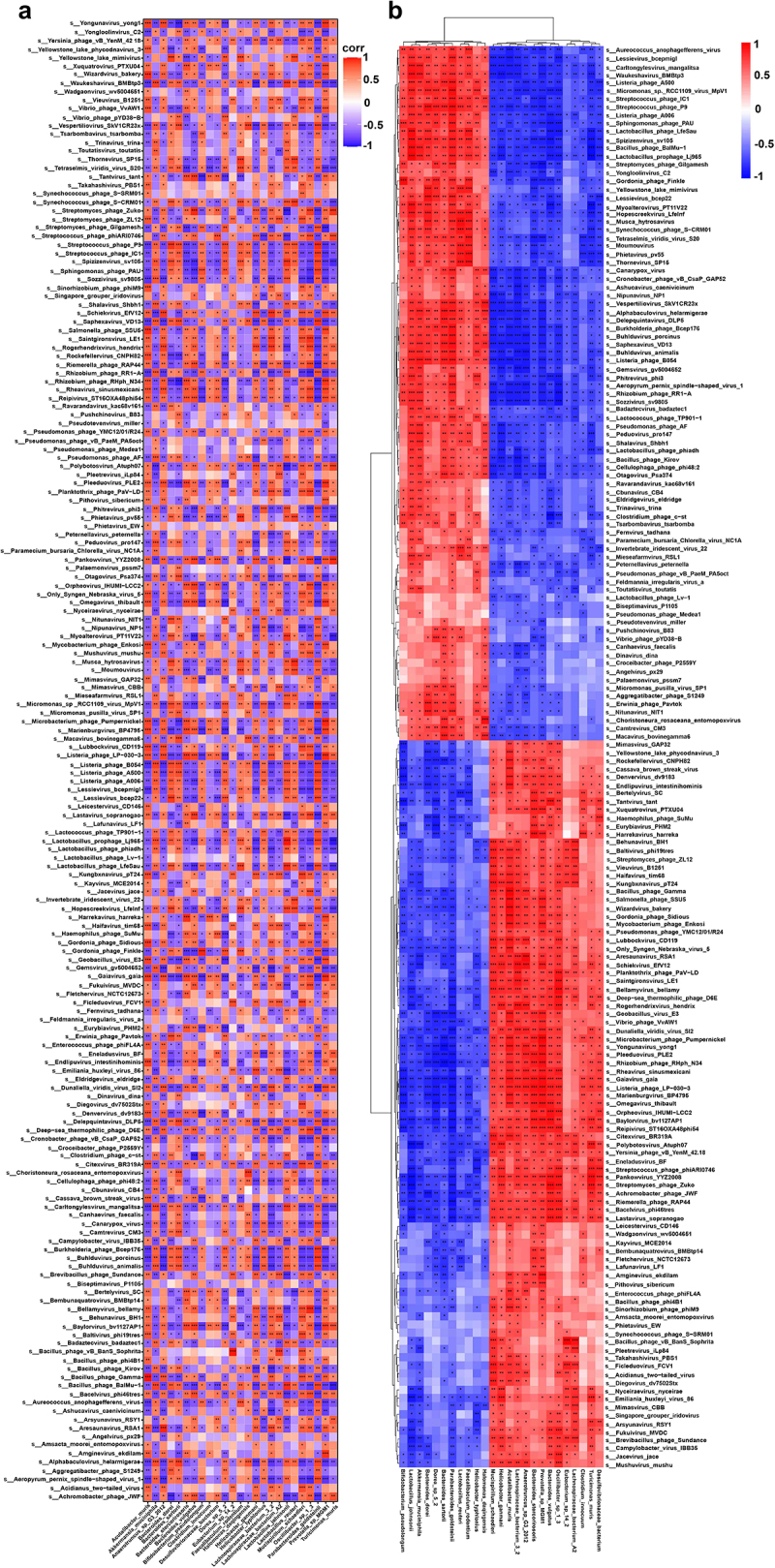
Association analysis between gut virome and bacteriome.

As mentioned above, the specific bacterial enrichments within CT and OP groups have been identified; we continued to explore the complex correlations between these bacteria and particular gut viruses, which are always neglected in microbiome studies. We observed that viruses such as *Phitrevirus_phi3*, *Listeria_phage_A006*, and *Spizizenvirus_sv105* tend to co-occur with *Akkermansia_muciniphila*, *Lactobacillus_johnsonii*, *Lactobacillus_reuteri*, *Bifidobacterium_pseudolongum*, and *Helicobacter_typhlonius* in the CT group, suggesting a symbiotic relationship that could be contributing to the maintenance of a healthy gut environment and may protect against bone density loss. Conversely, in the OP group, viruses with *Baltivirus_phi19tres*, *Gordonia_phage_Sidious*, *Lastavirus_sopranogao*, *Polybotosvirus_Atuph07*, and *Riemerella_phage_RAP44* were positively associated with *Prevotella_sp_MGM1*, *Turicimonas_muris*, and *Helicobacter_ganmani*, indicating a potential pathogenic synergy that might exacerbate detrimental processes involved in OP pathogenesis. The relationship between *Lactobacillus_johnsonii*, *Lactobacillus_reuteri*, *Akkermansia_muciniphila*, and viruses such as *Lastavirus_sopranogao*, *Polybotosvirus_Atuph07*, as well as *Riemerella_phage_RAP44*, were of particular notable, as these associations in the CT group demonstrated that the exist of probiotics may contribute to gut homeostatic balance via inhibiting multiple viruses. Through the lens of these associations, our study brought forth evidence indicative of a complex interplay between the gut microbiota and the virome, which could be integral to understanding the etiology and progression of OP (Figure 5a,b).

(a and b) Covariance heatmap of gut virome and bacteriome associations between OP and CT groups.

### Association analysis of gut bacteriome and virome with bone histomorphometry index

**Figure 6.**
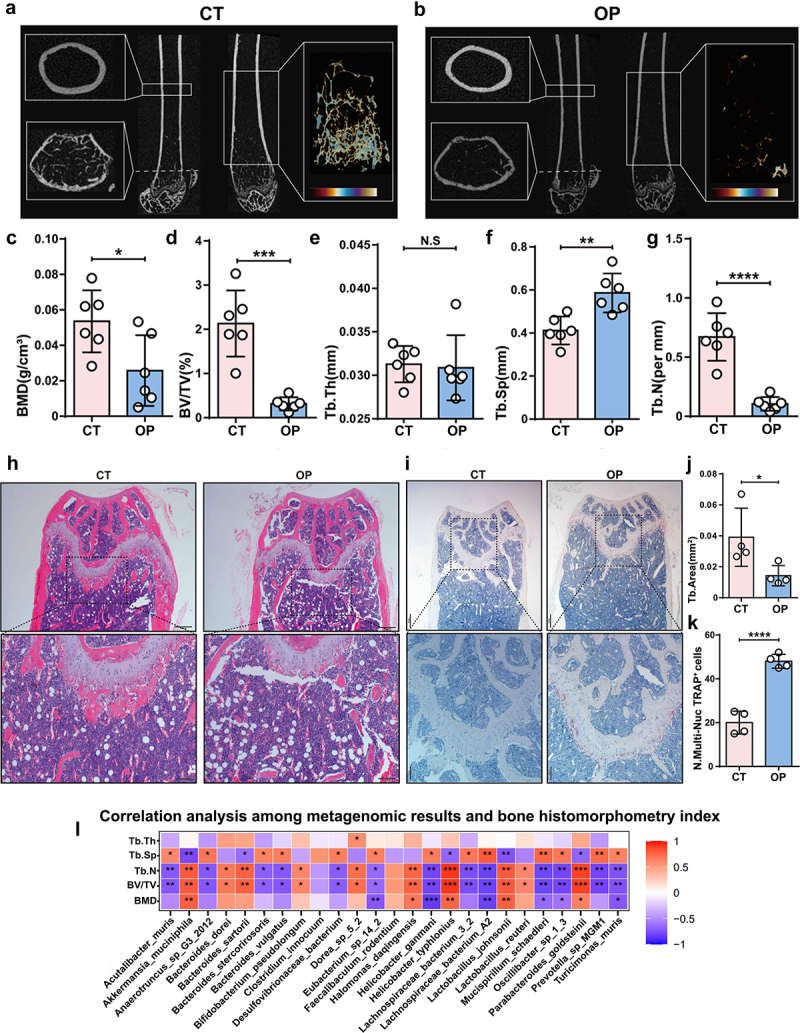
Association analysis of gut bacteriome with bone histomorphometry indexes.

As shown in Figure 6a,b, we developed femur longitudinal section outlines and contoured cortical bone from cross-section of µCT images. The representative µCT images of the cross-section and reconstructed trabecular structure at the distal femur demonstrated significant bone mass loss in the OP group. Quantification analyses of µCT results suggested that OVX surgery resulted in lower BMD (*p* = 0.0282), BV/TV (*p* = 0.0002), Tb.N (*p* < 0.0001) and higher Tb.Sp (*p* = 0.0032), with imperceptible Tb.Th (*p* = 0.8931) (Figure 6c–g). Additionally, H&E staining indicated that the OP group exhibited dramatically decreased bone area per slice, and TRAP staining demonstrated that osteoclast activities were dramatically enhanced after OVX surgery (Figure 6h–k). Additionally, we detected changes in osteoblasts at the distal femur between the OP and CT groups using two osteogenic markers, Osteocalcin (OCN) and Bone Morphogenetic Protein 2 (BMP2). Our results indicated no significant differences in the expression of OCN and BMP2 between the OP and CT groups, suggesting similar osteoblast activity in the osteoporotic condition (Fig. S4). However, this result may be due to the small sample size (only three samples in each group), which could have prevented us from observing a more definitive trend. Future studies with larger sample sizes are needed to further validate these findings (Fig. S4).

(a) Representative micro-CT images of longitudinal section femurs, cross-sectional view of the distal femurs, and reconstructed trabecular structure of the ROI in the CT group.

(b) Representative micro-CT images of longitudinal section femurs, cross-sectional view of the distal femurs, and reconstructed trabecular structure of the ROI in the OP group.

(c-g) Quantitative analyses of BMD, BV/TV, Tb.Th, Tb.Sp and Tb.N. (h and i) Representative femur images of H&E and TRAP staining between OP and CT groups.

(j and k) Quantitative analysis of H&E and TRAP staining.

(l). Association heatmap of different bacterial species with bone histomorphometry index.

To further investigate the intricate relationship between the gut microbiota, virome, and bone health, we performed correlation analyses between bone histomorphometry indexes and metagenomic results as well as viromic results. We revealed that *Akkermansia_Muciniphila*, *Halomonas_daqingensis*, *Helicobacter_typhlonius*, *Lactobacillus_johnsonii*, and *Parabacteroides_goldsteinii* were positively associated with BMD, BV/TV, Tb.N and negatively correlated with Tb.Sp. In specific, *Akkermansia_Muciniphila*, as a promising probiotic, is known for its beneficial effects on bone by tightening the intestinal barrier to ameliorate systemic inflammation.^28^ *Lactobacillus johnsonii* is a new probiotic strain and potential anti-inflammatory bacterium to treat a wide range of illnesses including obesity, diabetes, and colitis, which may possess a potential bone-protective role.^25^ These results contributed to the exploration of more probiotics, which may modulate bone metabolism by influencing nutrient absorption, and immune responses, or impacting gut permeability and inflammation. On the other hand, bacteria like *Eubacterium_sp_14_2*, *Helicobacter_ganmani*, *Mucispirillum_schaedleri*, *Oscillibacter_sp_1_3*, and *Turicimonas_muris* showing negative correlations with BMD and other bone indices might contribute to bone degradation processes or poor bone health due to factors such as dysbiosis-induced inflammation or by interfering with the host’s nutrient absorption essential for bone maintenance (Figure 6l).

**Figure 7.**
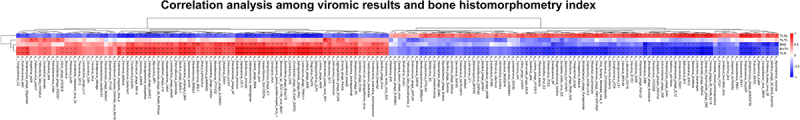
Association analysis of gut virome with bone histomorphometry index.

The correlation analysis between bone histomorphometry indexes and the gut virome has identified an intriguing positive association with certain viral strains, such as *Aeropyrum_pernix_spindle−shaped_virus_1*, *Alphabaculovirus_helarmigerae*, various *Bacillus_phages* like *BalMu −1* and *Kirov*, *Buhlduvirus* species, *Burkholderia_phage*, and phages infecting *Clostridium*, *Lactobacillus*, *Listeria*, *Pseudomonas*, *Streptococcus*, amongst others. These findings suggested a complex interaction between the gut virome and bone health, where certain bacteriophages and eukaryotic viruses may exert a positive influence on bone density and integrity. This could be related to the modulation of gut bacterial communities that in turn produce metabolites important for bone health, or by influencing host immunity in a way that favors bone formation or preservation. The presence of bacteriophages specific to probiotic bacteria, such as those infecting *Lactobacillus*, could be indicative of a dynamic interaction where the controlled lysis of such bacteria releases beneficial substances or regulates the gut environment favorably for bone health. On the other hand, we continued to analyze the diverse set of viral strains showing a negative correlation with BMD and other bone health indicators. We categorized these virus strains based on their hosts and potential impact on bone health. Bacteriophages targeting commensal bacteria such as *Achromobacter_phage_JWF*, *Bacillus_phage_vB_BanS_Sophrita*, *Brevibacillus_phage_Sundance*, *Riemerella_phage_RAP44*, *Streptococcus_phage_phiARI0746*, and *Streptomyces phages (ZL12, Zuko)* may indicate that these phages disrupt bacterial populations that play a role in maintaining gut health, which in turn could influence the gut-bone axis. Eukaryotic viruses infecting host cells or other microorganisms including *Singapore_grouper_iridovirus* and *Emiliania_huxleyi_virus_86* may activate host immune responses or cause chronic low-grade inflammation, which might lead to increased bone resorption and decreased BMD. Viruses infecting extremophiles or environment-specific organisms such as *Deep-sea_thermophilic_phage_D6E* and *Dunaliella_viridis_virus_SI2* were less directly related to human health, their presence and negative correlation may represent a broader environmental or ecological disturbance that could be associated with adverse host health outcomes (Figure 7). The negative correlations observed between these viral strains and bone health indicators suggested that perturbation of the virome can negatively impact bone health. There could be a complex interplay between altered microbial dynamics due to viral infections, the host’s immune response, and direct effects on bone cells leading to impaired bone formation or enhanced degradation. These findings underscored the importance of maintaining virome stability and integrity for potential preventative or therapeutic interventions targeting OP.

## Discussion

It is well known that gut microbiota widely participates in OP progression; however, the role of gut virome is poorly understood. Here, we mapped the profiles of the gut virome in OVX-induced OP and healthy controls, which demonstrated a marked alteration characterized by differential composition and structure, as well as loss of multiple viral functions. Furthermore, by integrating metagenomic shotgun sequencing, we demonstrated a close association between gut bacteria and viruses, uncovering their coordinated alterations and intricate interplay. This leaded to a compelling question of whether the viral communities in the gut are implicated in the progression of OP.

Previous studies have revealed the markedly altered gut virome in multiple metabolic diseases such as obesity, type 2 diabetes, and metabolic syndrome, which demonstrated that intestinal viruses may be involved in the development of metabolic diseases.^29,30^ OP is one of the most common metabolic bone diseases. In our study, we identified 174 significantly changed virus strains in OVX subjects; however, there is minimal understanding regarding the precise function of these viral indicators in the progression of OP. Indeed, bone homeostasis is regulated by a network of multiple cell types within the basic multicellular unit (BMU), including osteoclasts, osteoblasts osteocytes, bone lining cells, osteomacs, and vascular endothelial cells.^31^ The regulation of bone homeostasis by the gut virome encapsulates a multifactorial process that intertwines with immune modulation, microbial metabolism, and the complex interplay between the host and its resident viruses. Within the gut ecosystem, bacteriophages exert selective pressure on bacterial communities, potentially altering the balance of bone-modulating compounds such as SCFAs, which are known to influence bone metabolism. We observed that *Lactobacillus reuteri* which participates in the production of SCFAs was significantly decreased in the OVX group, which may be related to the alteration in related bacteriophage. SCFAs, such as butyrate and propionate, have been shown to modulate bone cell activity. For instance, butyrate can enhance osteoblast differentiation and bone formation while suppressing osteoclast activity, thus maintaining bone density.^32,33^ Additionally, these viral entities could also directly impact bone homeostasis by modulating the host immune response, as immune cells infiltrating the bone can affect corresponding osteoclast-resorptive and osteoblast-formative processes. Moreover, the gut virome can directly affect the bone microenvironment by altering the gut microbiota, thereby modulating bone lining cells and vascular endothelial cells. These changes can influence the secretion of extracellular matrix proteins and the regulation of blood flow, essential for bone health. Osteocytes, the primary mechanosensory cells, may also be affected by changes in the gut virome through altered cytokine production and growth factor secretion, influencing their role in bone maintenance and repair.

In addition, the significant downregulation of bacterial functions related to key metabolic processes, as revealed through the Pfam protein functions heatmap, indicated a systemic disruption within the gut ecosystem that may contribute to OP pathogenesis. These alterations in the bacterial landscape could diminish nutrient absorption and waste elimination, potentially compromising skeletal health through disrupted connections between gut microbiota balance and bone metabolism. Simultaneously, the gut virome showed functional changes, characterized by a significant decrease in vital activities necessary for viral replication and the production of new virions. Conversely, certain viral functions, including those related to amino acid biosynthesis and energy metabolism, were enhanced. These dichotomous shifts in virome dynamics underscored a potential reconfiguration of the viral community, which could have downstream effects on the microbiota-virome balance, immune system modulation, and, consequently, the integrity of skeletal structures.

Association between specific gut bacteria and their corresponding viral partners in CT and OP groups suggested that a well-balanced gut microbiota is crucial for bone health. The positive associations between probiotics like *Akkermansia muciniphila* and *Lactobacillus* species with beneficial viruses in the CT group reinforced the notion of a symbiotic relationship where microorganisms together with their bacteriophages may create a gut environment conducive to bone health preservation. This symbiosis could be underpinned by their roles in reducing inflammation and enhancing gut barrier function, thereby preventing systemic inflammatory processes that are known to contribute to bone density loss. The OP group demonstrated a contrasting microbial and viromic profile, with bacterial and viral strains showing potential pathogenic synergy. This aligned with the hypothesis that gut dysbiosis and an altered virome may play a role in exacerbating inflammation and bone resorption in OP. Crucially, our research indicated that the gut microbiota and the virome do not operate in isolation but are interdependent, impacting the gut-bone axis. The gut viruses may affect the composition of microbial communities through selective bacterial host lysis, modulating the availability of pro- and anti-inflammatory bacterial metabolites that influence bone metabolism. Eukaryotic viruses may further complicate this picture by modulating the host’s immune system, potentially impacting osteoclast and osteoblast activities.

The observed associations between the composition of gut bacterial and viral communities with markers of bone structure suggested that both the gut microbiome and virome might be critical elements that could be harnessed in the development of upcoming treatment approaches. Probiotic supplementation could perhaps be tailored based on an individual’s gut virome to optimize beneficial effects on bone health. For instance, a recent study demonstrated that the relative abundance of *Prevotella histicola* was significantly decreased in OVX mice, and *Prevotella histicola* supplementation could perform protective effects on bone mass through its modulation of gut permeability as well as its inhibitory effects on osteoclast activity.^34^ The interactions between *Prevotella histicola* and the gut virome could be pivotal, as specific bacteriophages may regulate its abundance and activity, potentially enhancing its protective effects on bone health. However, we must be cautious and recognize the complexity of these systems. The observed associations do not necessarily imply causation, and therefore experimental validation through animal models and clinical trials would be required to ascertain causative relationships and therapeutic potential. In conclusion, our study underscored the intricate and potentially influential interactions between the gut microbiota, the virome, and bone homeostasis. These findings offered novel perspectives on the interactions between gut and bone health and laid the groundwork for subsequent studies into the manipulation of the microbiome and virome as a potential tactic in the prevention and management of OP.

## Materials and methods

### Establishment of OVXinduced osteoporotic mouse model and collection of fecal samples

All experimental mice applied in the study were 8-week-old C57BL/6J female mice (weighting 20–22 g) from the Hunan SJA Laboratory Animal Co., Ltd. All experimental procedures involving these mice were conducted in compliance with the guidelines for the ethical treatment and use of laboratory animals and were approved by the Ethical Review Board of the Army Medical University (No. AMUWEC20232385). All mice were raised under specific pathogen-free (SPF) conditions.

The mice were anesthetized with pentobarbital sodium (50 mg/kg), ensuring that they were unconscious and experienced no pain during the operation. A small incision was made on each side of the back to provide access to the ovaries. The ovaries were carefully located and dissected from the surrounding tissues, taking caution to identify and manage any blood vessels to minimize bleeding. The incision sites were meticulously sutured to promote proper wound healing after surgery. In the control group of OVX mice, a similar surgical procedure was performed as in the OVX group, but without the removal of the ovaries. There were six mice in CT group and six mice in OVX group.

Stool samples were gathered on day 56 post-operation into sterile 1.5 ml tubes. Each individual mouse was placed in a sterile mouse cage without bedding, allowing the mouse to defecate naturally. The feces were then collected using sterile tweezers and placed into a 1.5 mL sterile EP tube. For each new sample collection, the tweezers were replaced to ensure sterility. This process was repeated until feces from all mice were collected. The tubes containing the feces were immediately stored at −80°C to preserve the samples. Each fecal sample weighed approximately 200 mg. A set of six specimens from the OVX mouse cohort and another sextet from the CT mouse cluster were obtained and preserved in a deep-freeze storage unit, pending further analysis.

### Micro-Computed Tomography (CT) analysis

Femurs from mice in OVX and CT groups were collected and preserved in 4% paraformaldehyde (PFA) overnight. Subsequently, they were subjected to micro-CT analysis using the Bruker Skyscan 1272 system (Kontich, Belgium), following the methods outlined in previous studies.^35^ The micro-CT scanner utilized a 60 kV X-ray tube with settings of 166 μA and 1700 ms integration time, and the isotropic voxel size was set at 7.0 μm. Reconstruction of images utilized the NRecon application (Version 1.6.10, from Kontich, Belgium), with subsequent data evaluation carried out via analytical tools (Version 1.15.4.0) sourced from the same producer. Moreover, for examining the attributes of trabecular bone in the distal femur metaphysis, a 3D visualization platform (μCT Vol v2.2) was employed.

In assessing the trabecular bone structure, a specific region of interest (ROI) was determined, specifically located 1.0 mm proximal to the femoral growth plate and extending 4.0 mm in length. This was done while omitting the growth plate and primary spongiosa. Trabecular structures underwent thresholding within a range of 86 to 255 on the 8-bit grayscale spectrum. Assessments were conducted to establish values for variables such as bone mineral density (BMD), trabecular separation (Tb. Sp), the ratio of bone volume to total volume (BV/TV), trabecular bone number (Tb. N), and trabecular thickness (Tb. Th). The quantitative data obtained reflected the characteristics of the bones in the different study groups and were analyzed to draw relevant conclusions from these indicators.

### Histological analysis

Histological analysis was performed on the femurs collected from mice in the OVX and CT groups. The femurs were fixed in 4% PFA and then subjected to decalcification with 10% ethylenediaminetetraacetic acid (pH = 7.2). Subsequently, the femurs were embedded in paraffin and sectioned into 5 µm thin slices using a microtome. The slices were cleared of paraffin with the aid of xylene and subjected to progressive rehydration by employing descending concentrations of alcohol. Following this, the slices underwent a staining process utilizing Hematoxylin and Eosin (HE) as well as Tartrate-resistant Acid Phosphatase (TRAP) staining techniques.

### Immunohistochemistry (IHC) analysis

For immunohistochemical staining, femoral sections were incubated with the corresponding primary antibodies including anti-BMP2 (Boster, Cat#: BA0585) and anti-Osteocalcin (Boster, Cat#: PB1008) overnight at 4°C. Subsequently, they were incubated with the corresponding secondary antibodies (Santa Cruz, CA) for 1 h. The relative expressions of positive staining were evaluated by Image J software.

### DNA extraction and metagenomic shotgun sequencing

DNA from fecal samples was isolated utilizing the sodium dodecyl sulfate (SDS) technique. The isolated DNA was then diluted to a concentration of 1 ng/μL with sterile double-distilled H2O for subsequent analysis. The quality and purity of DNA, in terms of its degradation and possible contaminants, were evaluated on 1% agarose gel electrophoresis. The DNA purity, judged by the optical density ratio (OD260/OD280), was quantified with a NanoDrop Microvolume Spectrophotometer provided by Thermo Fisher Scientific, based in the US. The DNA concentration was determined using the Qubit® double-stranded DNA (dsDNA) Assay Kit, executed on a Qubit® 2.0 Fluorometer (Life Technologies, positioned in Carlsbad, CA, USA). Sequencing of all the samples was conducted using the Illumina sequencing platform, utilizing the paired-end 150 (PE150) approach. Removal of adaptors and exclusion of reads of low quality were executed by the application of trimmomatic-0.39 software. To eliminate host-derived sequences, the software SOAP2 (version 2.20) was used to align sequences to the reference host genome (GRCm39), discarding matches with over 90% similarity. Taxonomic distribution within the metagenomic data was determined with MetaPhlAn2, which exploits clade-specific markers for broad-range microbial classification (including bacteria, archaea, viruses, and eukaryotes) down to the species tier, employing the software’s standard settings. Concurrently, the refined reads were mapped to the updated reference catalog of gut microbiome genes via SOAP2 (version 2.20), setting a minimum matching threshold of 90% identity and a minimum read length coverage of 95%. Abundance profiles for genes were generated as outlined in prior methodology. To deduce functional profiles, the respective gene abundances were tallied according to their corresponding Kyoto Encyclopedia of Genes and Genomes (KEGG) orthologous (KOs) categories.

### Viral metagenomic analysis

We performed viral metagenomic analysis on paired-end sequenced samples using the Illumina platform (insert size 350bp, 2 × 150 nt paired-end sequencing). The samples underwent quality assessment with FastQC (v0.11.9) and Trimmomatic (v0.36) for sequence trimming and adapter removal. Human DNA was subtracted using soap2.22 (−c 0.9) referencing the hg38 genome. Viral contigs were assembled with SPAdes (v3.11) and screened via VirSorter2 (v2.1, score > 0.95 and not lt2gene) and VirFinder (v1.1, score > 0.7 and p-value <0.05) to eliminate bacterial-like sequences, followed by CD-HIT-EST (v.4.8.1, -c 0.95 -d 0) for clustering at 95% identity. Sequences of viral contigs were queried using blastn (version 2.11.0) against viral genomic databases in RefSeq, applying a threshold for the expectation value (E value) of less than 1e-10. To categorize the top five matching results, taxonkit (version 0.7.2) was used in conjunction with the NCBI taxonomic data to identify the lowest common ancestor (LCA), which was then assigned to the respective contigs. Contigs that remained unclassified from this process were subjected to further examination based on their open reading frames (ORFs). Prediction of ORFs present in these contigs was carried out with the Prodigal tool (version 2.6.3, deploying the -p meta option) specifically for the analysis of viral DNA sequences.

### Statistical analysis

We conducted a two-tailed Wilcoxon rank-sum test to evaluate the disparity in α diversity, and a permutation multivariate analysis of variance (PERMANOVA) was utilized to assess the disparity in β diversity among the groups being compared. An analysis of Spearman’s rank correlation was carried out for the association between features that exhibited differential enrichment. Except where noted differently, all the statistical tests were performed using the R programming environment, and *p* values below 0.05 were interpreted as denoting statistically significant differences. Other information on the statistical analyses utilized is specified in the captions accompanying figures and included in the results where appropriate.

## Supplementary Material

Supplemental Material

## Data Availability

The data that support the findings of this study are available from the corresponding author upon reasonable request.
