# Supplementary material for: Alterations of gut virome with close interaction in the progression of estrogen deficiency-induced osteoporosis
Source: Gut Microbes. 2024 Dec 9;16(1):2437250. doi: 10.1080/19490976.2024.2437250 (PMC11633233; doi:10.1080/19490976.2024.2437250)
Supplement: Supplemental Material [file KGMI_A_2437250_SM4251.docx]

**Supplementary Materials**


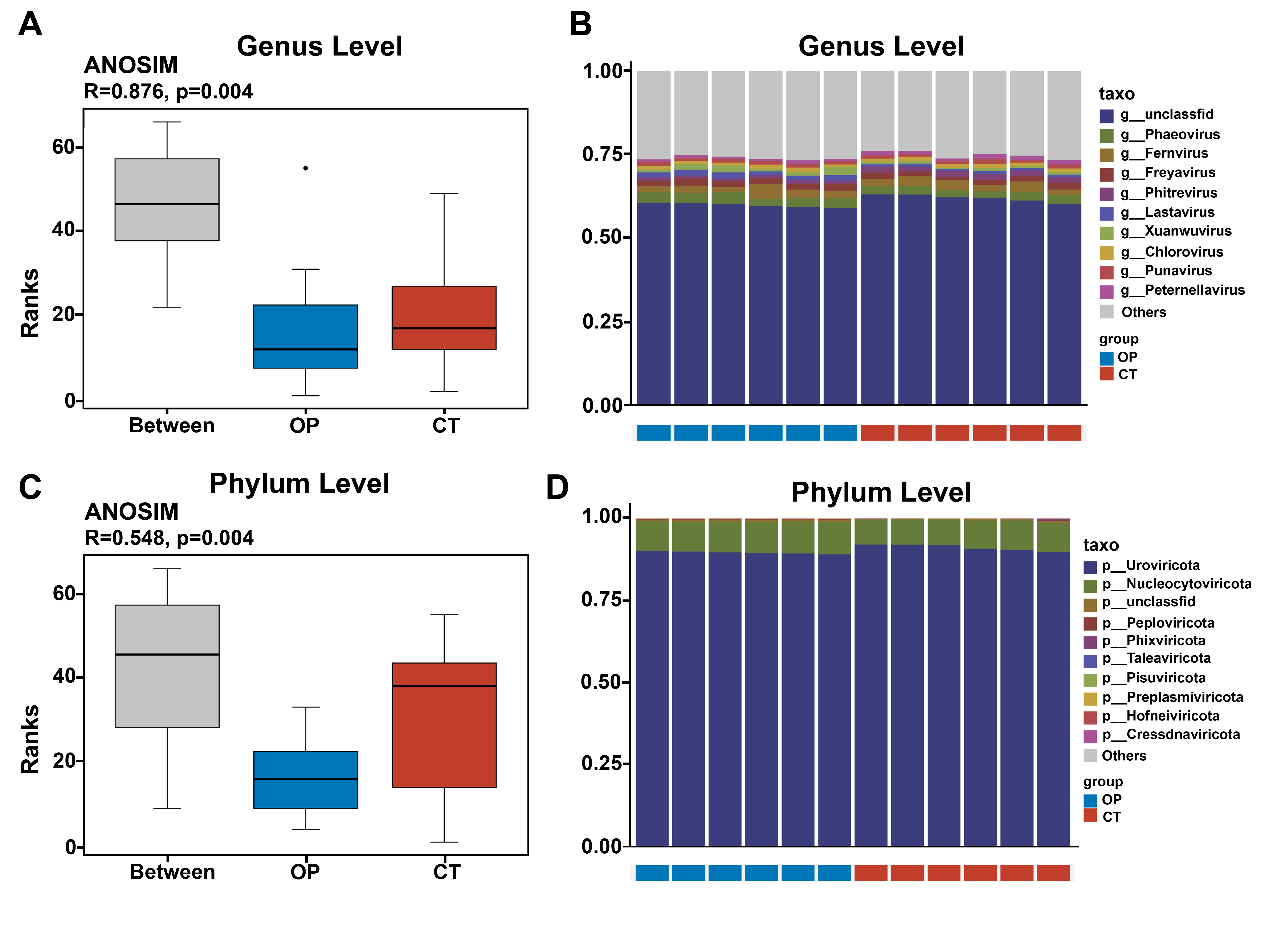


**Fig. S1** Gut virome distribution between OP and CT group at genus and phylum levels.

1. Analysis of similarities at the genus level.
2. Bar graphs of the virome at the genus taxonomic level between OP and CT groups.
3. Analysis of similarities at the phylum level.
4. Bar graphs of the virome at the phylum taxonomic level between OP and CT groups. The relative abundance of each sample was plotted.


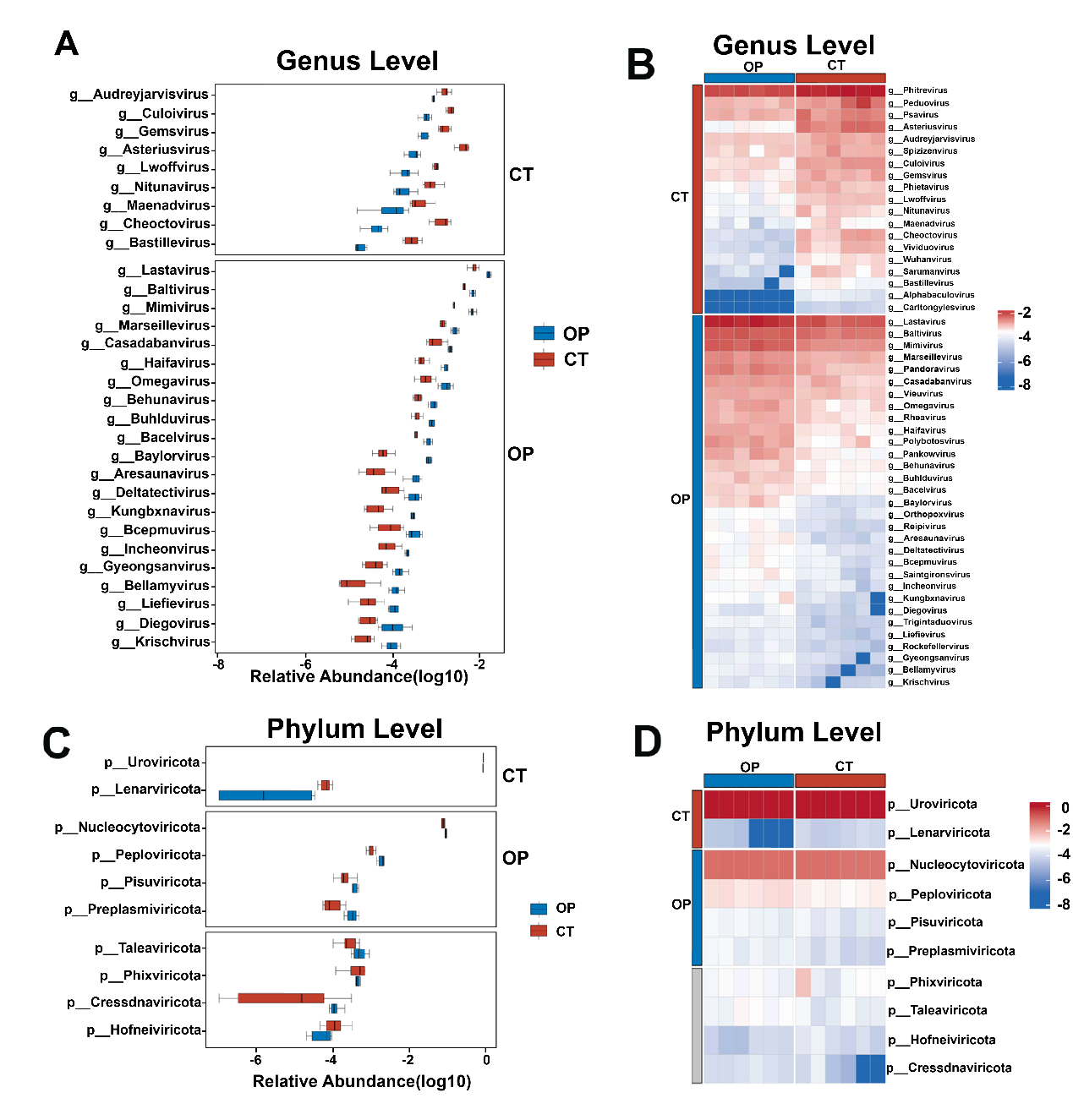


**Fig. S2** Differential virus distribution between OP and CT groups at genus and phylum levels.

1. Statistical differences of gut virome at the genus taxonomic level between OP and CT groups were evaluated with box plots.
2. Heatmap of different gut virome at genus taxonomic level between OP and CT groups. Color in the heatmap was utilized to describe specific general abundance, with blue indicating lower abundance and red indicating higher abundance.
3. Statistical differences of gut virome at the phylum taxonomic level between OP and CT groups were evaluated with box plots.
4. Heatmap of different gut virome at phylum taxonomic level between OP and CT groups. Color in the heatmap was utilized to describe specific phylum abundance, with blue indicating lower abundance and red indicating higher abundance.


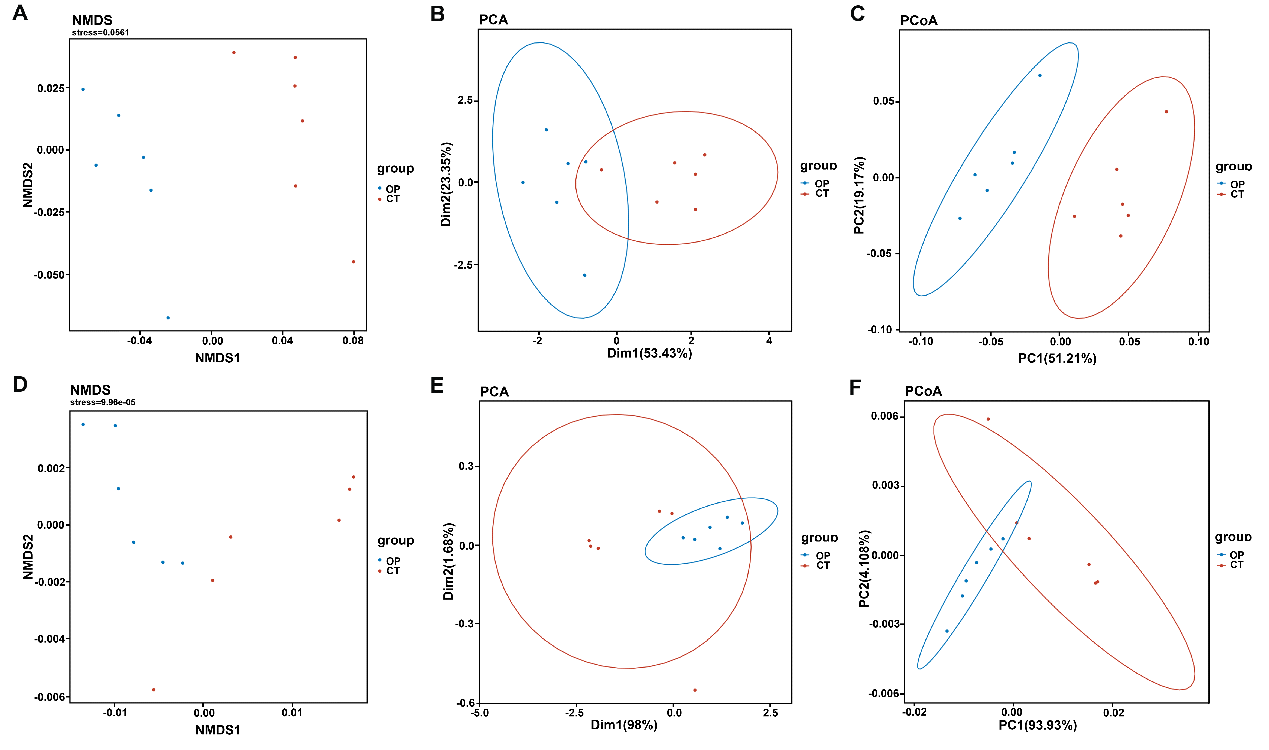


**Fig. S3** Differential viral profiles between OP and CT groups.

(A-C) The analyses of Non-metric Multidimensional Scaling, PCA, and PCoA at the genus level between OP and CT groups.

(D-F) The analyses of Non-metric Multidimensional Scaling, PCA, and PCoA at the phylum level between OP and CT groups.


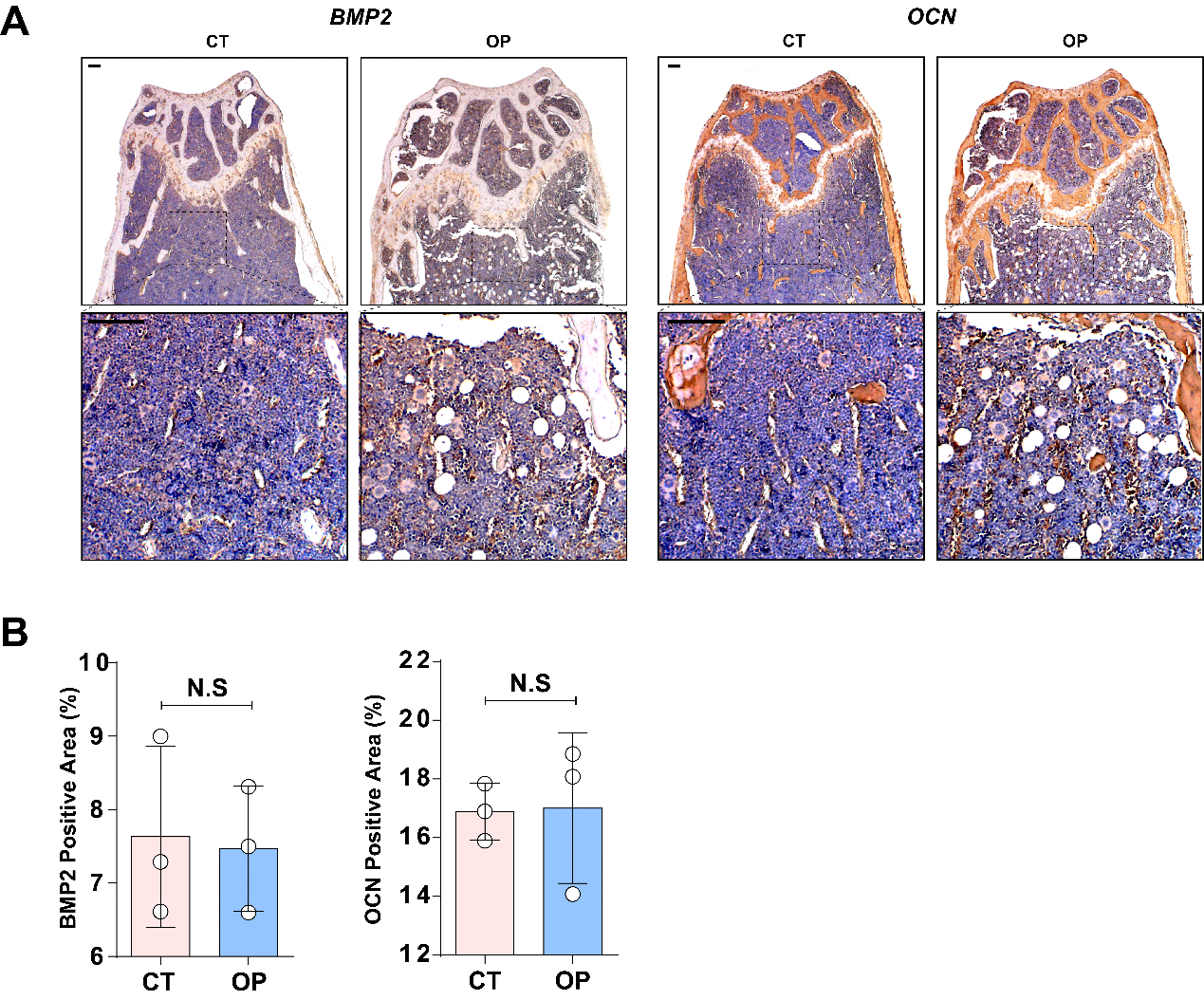


**Fig. S4** The evaluation of osteoblast activity in femurs of OP and CT groups.

(A) Immunohistochemical staining of BMP2 and OCN in femurs of OP and CT groups.

(B) Quantitative evaluation of BMP2 and OCN staining.
